# Delayed diagnosis and issues with pump usage are the leading causes of diabetic ketoacidosis in children with diabetes living in Newfoundland and Labrador, Canada

**DOI:** 10.1186/s13104-015-1115-y

**Published:** 2015-04-16

**Authors:** Jessica Jackman, Roger Chafe, Daniel Albrechtsons, Robert Porter, Colleen Nugent, Shahzad Waheed, Leigh Anne Newhook

**Affiliations:** Janeway Pediatric Research Unit, Memorial University, 300 Prince Philip Drive, St. John’s, A1B3V6 NL, Canada; Janeway Children’s Health and Rehabilitation Centre, Eastern Health, St. John’s, A1B3V6 NL, Canada

**Keywords:** Diabetic ketoacidosis, Type 1 diabetes, Pediatric, Prevention

## Abstract

**Background:**

Newfoundland and Labrador (NL) has a very high incidence of type 1 diabetes (T1DM) and admission rate for diabetic ketoacidosis (DKA). The purpose of this study was to identify characteristics and precipitating factors associated with pediatric DKA in this population.

**Methods:**

This was a retrospective study on children diagnosed with DKA from 2007–2011 admitted to the province’s only tertiary care pediatric hospital. Demographics, biochemical characteristics, and reasons for DKA diagnosis were analyzed. Chi-square and Fisher Exact tests were performed for categorical variables; t- and non-parametric Kruskal-Wallis tests were performed for continuous variables.

**Results:**

A total of 90 children were admitted with DKA (39.5% newly diagnosed; 60.5% were previously diagnosed). The rate of DKA on presentation for incident cases was 22.1%. More severe cases of DKA occurred in younger, newly diagnosed patients. Almost half of preexisting diabetes cases were recurrent DKA (49.1%). The most common presenting characteristics of newly diagnosed patients were weight loss, bedwetting, polyuria, polydipsia, and neurologic symptoms. Pre-existing diabetes patients most often presented with abdominal pain and vomiting. Diagnosis of diabetes in new patients and issues related to interrupted insulin delivery in pre-existing patients using insulin pump therapy were the most common factors associated with DKA. Of the newly diagnosed patients presenting in DKA, 64% had seen a physician in the weeks leading up to diagnosis.

**Conclusions:**

Pediatric patients have predictable patterns associated with a diagnosis of DKA. Most cases of DKA could be prevented with earlier diagnosis and improved education and problem-solving by families and health care providers. DKA preventative strategies are recommended and should be aimed at patients, their families, and health care professionals especially those outside of pediatric centers.

## Background

In Canada, type 1 diabetes mellitus (T1DM) is one of the most common chronic diseases affecting the pediatric population. In 2008/09, the Canadian Chronic Disease Surveillance System identified over 3,000 new cases of pediatric diabetes [1]. Newfoundland and Labrador (NL) has one of the highest recorded incidences of T1DM worldwide [2,3] and a very high hospitalization rate for diabetic ketoacidosis (DKA) [4]. Among children diagnosed with T1DM, diabetic ketoacidosis (DKA) is the leading cause of morbidity and mortality [5]. DKA is more typically associated with T1DM, however it can also occur in youth with T2DM [6].

In those with established diabetes, insulin omission, whether deliberate or unintentional, is the most common precipitating factor for DKA. In new-onset diabetes, a missed or delayed diagnosis of diabetes is most often an issue [7]. Understanding the factors associated with the development of DKA in the pediatric population is vital in order to prevent DKA, especially in a region with a very high incidence of T1DM and DKA admissions.

## Methods

This was a retrospective study of all patients in NL under the age of 18 admitted with DKA to the only tertiary care pediatric hospital (Janeway Child Health Care Centre, Eastern Health, St. John’s, NL) during 2007–2011. Patients included met the biochemical criteria outlined in the ISPAD Clinical Practice Consensus Guidelines [8].

### Data collection and analysis

All charts with the ICD codes for diabetes or DKA were included. Data was collected by a pediatric research nurse using a data abstraction tool that was piloted and revised for this study. Recorded data included patient demographics, biochemical parameters, and whether the patient had been previously diagnosed with diabetes. In those with pre-existing diabetes, data collected included the method of insulin treatment, most recent A1C, any previous admissions of DKA, presenting symptoms, documented reasons for DKA and whether the patient had been transferred from a community hospital. Criteria for infection included documentation by a physician in the chart as the reason for DKA.

### Definitions of DKA

Mild (pH < 7.3; bicarbonate < 15 mmol/L); moderate (pH < 7.2; bicarbonate < 10 mmol/L) severe (pH < 7.1; bicarbonate < 5 mmol/L).

### Statistical analysis

Associations between key variables and DKA severity were examined. Chi-square and Fisher exact tests were performed for categorical variables; t- and non-parametric Kruskal-Wallis tests were performed for continuous variables. A p value of <0.05 was considered statistically significant.

### Ethics

Approvals were obtained from the NL Human Research Ethics Authority (HREA) according to the Declaration of Helsinki, the Janeway Children’s Health and Rehabilitation Centre where the study was performed, and the Eastern Health Research Proposal Approval Committee (RPAC).

## Results

### Demographic characteristics

90 hospital admissions met the diagnostic criteria for DKA. One patient had T2DM, the rest were T1DM. 39.5% were newly diagnosed and 60.5% were previously diagnosed. There were 163 children with newly diagnosed diabetes during the timeframe of the study; of these 36 (22.1%) presented with DKA. Cases of DKA were more likely to be female (f = 60%, m = 40%). The mean age of females presenting in DKA was higher than males (f = 12.2 years, m = 9.7 years; p = 0.03). Patients with pre-existing diabetes were older at presentation of DKA than newly diagnosed patients (newly diagnosed 6.6 years, pre-existing diabetes 14.0 years; p < 0.01). Method of treatment for pre-existing diabetes (not in table): 58.5% with an insulin pump; 39.6% via insulin by injection, 1 adolescent patient on metformin and insulin. 49.1% of previously diagnosed of cases were recurrent DKA; less than 1% had ≥ 2 previous episodes of DKA. Pre-existing patients treated with insulin by injection were more likely to have more severe DKA than patients treated with an insulin pump (83.3% versus 16.1%; p < 0.006) (Table 1).

### Biochemical parameters at presentation of DKA

There were 38.9% of cases classified as mild DKA, 32.2% as moderate and 18.9% as severe. Degree of acidosis was more severe in newly diagnosed patients compared to pre-existing diabetes based on both mean bicarbonate (p < 0.03) and base excess concentrations (p < 0.01). Potassium concentrations were lower in females versus males (f = 4.8 mmol/l, m = 5.3 mmol/l; p = 0.04).

### Symptoms and presentation of DKA

For newly diagnosed patients, the most common symptoms were weight loss (100%), nocturnal enuresis and/or nocturia (100%), polyuria (79.5%), polydipsia (72.3%) and CNS symptoms such as altered level of consciousness (66.7%). For pre-existing diabetes, the most common presenting symptoms were vomiting (74.6%) and abdominal pain (69%). 64.1% of newly diagnosed patients presenting in DKA had been assessed by a primary care physician in the weeks prior to admission. For pre-existing diabetes patients, 49.1% had been previously admitted for DKA. The most common reasons documented for DKA in previously diagnosed patients included insulin delivery interruption (e.g. kink in tubing, bent cannula, site issues/ lipohypertrophy) (23.6%), infection (20.0%), and non-adherence (14.5%) (Table 2).

### Hospitalization and length of stay (LOS)

**Table 1:** **Patient demographic and biochemical characteristics at diagnosis of DKA**

	**Males (n = 36)**	**Females (n = 54)**	**p**	**Newly diagnosed (n = 36)**	**Pre-existing patients (n = 55)**	**p**
**Age/years**	9.7 (4.8)	12.2 (5.4)	**0.03**	6.6 (5.1)	14.0 (3.0)	**<0.01**
**0-4 years**	2.2 (1.1)	1.3 (0.5)	0.10	1.7 (0.9)	**	*
**5-9 years**	7.5 (1.4)	7.3 (2.0)	1.00	6.6 (1.6)	8.4 (1.1)	**0.02**
**10-14 years**	11.7 (1.3)	12.7 (0.9)	**0.02**	12.0 (1.2)	12.2 (1.3)	0.62
**≥15 years**	16.3 (0.8)	16.5 (0.6)	0.80	17.0 (0.01)	16.4 (0.7)	0.29
**Mean A1C**	8.8 (1.2)	9.6 (1.7)	0.08	**	9.3 (1.6)	*
**pH**	7.2 (0.08)	7.2 (0.13)	0.47	7.1 (0.12)	7.2 (0.10)	0.11
**HCO3**	10.5 (5.4)	10.2 (5.4)	0.83	8.2 (5.1)	11.7 (5.1)	**0.03**
**Base excess**	−14.9 (7.2)	−16.3 (6.1)	0.33	−18.7 (7.1)	−14.0 (5.5)	**0.01**
**K mmol/l**	5.3 (1.2)	4.8 (0.76)	**0.04**	5.1 (1.4)	5.0 (0.75)	0.82
**Na mmol/l**	135.0 (5.7)	134.8 (3.79)	0.86	135.3 (6.7)	134.7 (2.9)	0.62

**Group contained no cases; *p-value not computed; All results are mean (SD).

**Table 2:** **Characteristics presenting symptoms of DKA and Length of Stay (in days), and time to DKA resolution**

	**New diabetes %**	**Pre-existing diabetes %**
Weight loss	100	0.0
Bedwetting	100	0.0
Polyuria	79.5	20.5
Polydipsia	72.3	27.7
Neurologic symptoms (Altered LOC or irritability)	66.7	33.3
Abdominal pain	31.0	69.0
vomiting	25.4	74.6
other	42.4	57.6
Treated at peripheral hospital prior to transfer	42.9	57.1
Saw MD days/weeks prior to admission for DKA	64.1	NA
Insulin pump	NA	56.4
Insulin by injection	NA	38.2
metformin	NA	2
previous admissions for DKA	0.0	49.1
LOS in PICU (mean # days)	1.0	0.44 (p* < 0.01)
LOS medical floor (mean # days)	5.0	1.0 (p* < 0.01)
DKA resolution (mean # hours)	13.5	7.5 (p* < 0.01)

NA = not applicable; *Statistical analysis performed using Mann–Whitney non-parametric test.

A positive gradient between severity of DKA, longer time to resolution of acidosis and longer length of stay (LOS) was observed. Newly diagnosed patients had a longer LOS as well as a longer time to DKA resolution. For patients transferred from outside facilities, 29% of cases were treated in community hospitals prior to presentation at the tertiary care hospital (42.9% were newly diagnosed patients; 57.1% had pre-existing diabetes) (Table 2).

## Discussion

This retrospective study covers a variety of presenting characteristics of pediatric DKA in a pediatric tertiary care center with a very high incidence and prevalence of pediatric diabetes. The rate of DKA on presentation for incident cases is 22.1% in this tertiary care hospital. Based on other regions, DKA diagnosis on presentation of new onset T1DM occurs about 15-67% of the time. Regions having higher rates of T1DM generally have lower rates of DKA at the time of diagnosis which is consistent with our findings [9]. This may be related to heightened awareness of the disease in areas with higher prevalence of pediatric diabetes.

Patients with diabetes are susceptible to DKA under stressful conditions, such as trauma, surgery, or infections. Worldwide, infection is the most common precipitating cause for DKA, occurring in 30-50% of cases. Other precipitating causes are psychological stress, sick-day mismanagement and non-adherence with insulin therapy [11]. The most common reasons given in this study for presenting in DKA in patients with pre-existing diabetes were problems with interruption of insulin delivery (e.g. kink in tubing, bent cannula, and cannula insertion site problems such as lipohypertrophy), infection and non-adherence. Other reasons included stress, travel-related issues (i.e. leaving insulin at home), poor metabolic control, changes to treatment regimen and sick day mismanagement. These reasons have been seen in other studies looking at risk factors for presenting in DKA [9-11]. The high rate of insulin delivery interruption problems seen in our study likely reflects the fact that more pediatric patients are using insulin pump therapy than found in earlier studies. Our center has high a percentage of insulin pump users (approximately 70% of patients). Other studies have shown psychiatric disorders, lower socioeconomic status and lack of appropriate health insurance as risk factors [12], which we did not identify in this study. This may be partially due to universal government-funded medical care availability for all residents.

Cases of DKA were more likely to occur in females in this study. This observation is consistent with other studies that have looked at gender differences in DKA [4,13]. We found that females had a higher mean A1C level which indicates poorer metabolic control. We did not look at specific reasons why DKA was more common in females or why they were older, but it has been suggested in other research that psychosocial issues, such as familial conflict and behavior problems, as well as intentional weight reduction associated with insulin omission have been linked with DKA [14,15].

Of the newly diagnosed patients, 50% met the criteria for either moderate or severe acidosis. Research suggests poorer long-term outcomes in patients who present in DKA [16]. Also, patients with a moderate or severe presentation had a longer length of stay in hospital, translating into higher costs associated with hospitalization. The most common presenting symptoms in the newly diagnosed patients included polyuria, polydipsia, weight loss, bedwetting and neurologic symptoms such as irritability. In those previously diagnosed, the most common symptoms of DKA were vomiting and abdominal pain. Understanding the most common presenting symptoms is important for improving early recognition and management of diabetes and DKA. In our study, although nearly two thirds of newly diagnosed patients had documentation in their medical chart that they had seen a health care professional with one or more of these symptoms in the weeks leading up to their admission for DKA, diabetes was not diagnosed. This trend has also been seen elsewhere [13]. Earlier recognition and diagnosis may have reduced morbidity and prevented ICU admission.

Many of the patients treated at our tertiary care center were transferred in from community hospitals (43% of newly diagnosed patients; 57% of previously diagnosed patients). It is important that health care professionals in regional and rural hospitals are knowledgeable about the diagnosis of diabetes, are familiar with newer technologies and the treatment of pediatric DKA.

Limitations: This was a retrospective study; therefore there is likely missing data and some factors maybe underrepresented because data was not collected in a prospective manner. This sample is also limited as it represents patients presenting to the only pediatric tertiary care facility for the province of NL, which services about 50% of the population. The utilization of a consecutive sample over a number of years serves to limit selection bias in this study. However, the single-center design does have the potential to introduce bias. A significant number of patients were transferred from other centers and some patients were treated at community hospitals. In this case, it is possible that the sample is biased toward a more severe and younger population as these cases may be more likely to have been transferred.

Main Findings and Recommendations:

1. Newly diagnosed diabetes: The symptoms of diabetes are very predictable in children. Parents, caregivers and health care providers require awareness of the signs and symptoms of diabetes to improve earlier diagnosis and prevention of DKA. In Parma, Italy, a teaching campaign reduced the number of patients presenting in DKA from 78% to 12.5% showing how public awareness campaigns can be effective [17]. Parma, however is a small geographic area and the challenges can be different in a vast geographic area with many primary care physicians.
2. Pre-existing diabetes: The causes of most cases are predictable and prevention of DKA in these patients is theoretically possible in almost all cases. Families and patients need education to understand how DKA occurs. Insulin pump delivery is a risk factor for DKA because it uses rapid acting insulin only. Families using this method of treatment need education specifically around problems with insulin delivery and the management of high blood sugars. Recurrent DKA is an issue for some patients and multidisciplinary treatment and preventative strategies that include psychosocial assessment should be tailored to those at risk.
3. Community hospitals play a significant role in the management of pediatric diabetes and DKA. Continued knowledge translation aimed at primary care providers who treat children and youth are important, especially as newer technologies become available. Resources must not be limited to larger centers as many of these patients present to smaller community health care facilities.

## Conclusions

Newly diagnosed and pre-existing patients have predictable patterns that are associated with a diagnosis of DKA. To address the issue of high rates of DKA in our region, the clinical and research teams have held educational sessions on DKA at hospitals across the province and have developed a nationally accredited course for primary care physicians. We have also developed a series of resources for families to help prevent DKA. Posters explaining the common symptoms of diabetes in children have been distributed to family physicians and public health clinics, pharmacies, and schools across the province. It is unclear yet what impact these interventions may have, but we will continue to measure DKA rates in the hope of identifying a decrease in DKA rates.

## References

[1] Public Health Agency of Canada. Report from the National Diabetes Surveillance System: Diabetes in Canada. 2009. URL http://www.phac-aspc.gc.ca/cd-mc/publications/diabetes-diabete/facts-figures-faits-chiffres-2011/chap6-eng.php

[2] Newhook L, Penney S, Fiander J, Dowden J (2012). Recent incidence of type 1 diabetes mellitus in children 0–14 years in Newfoundland and Labrador climbs to over 45/100,000: a retrospective time trend study. BMC Res Notes.

[3] Newhook LA, Grant M, Sloka S, Hoque M, Paterson AD, Hagerty D (2008). Very high and increasing incidence of Type 1 Diabetes Mellitus in Newfoundland and Labrador, Canada. Pediatr Diabetes.

[4] Alaghehbandan R, Collins KD, Newhook LA, MacDonald D (2006). Childhood type 1 diabetes mellitus in Newfoundland and Labrador, Canada. Diabetes Res Clin Pract.

[5] Dahlquist G, Källén B (2005). Mortality in childhood-onset type 1 diabetes: a population-based study. Diabetes Care.

[6] Sellers EA, Dean HJ (2000). Diabetic ketoacidosis: a complication of type 2 diabetes in Canadian aboriginal youth. Diabetes Care.

[7] Wolfsdorf JI (2014). The International Society of Pediatric and Adolescent Diabetes guidelines for management of diabetic ketoacidosis: do the guidelines need to be modified?. Pediatr Diabetes.

[8] Wolfsdorf J, Craig ME, Daneman D, Dunger D, Edge J, Lee W (2009). Diabetic ketoacidosis in children and adolescents with diabetes. ISPAD Clinical Practice Guidelines 2009 Compendium. Pediatr Diabetes.

[9] Dunger DB, Sperling MA, Acerini CL, Bohn DJ, Daneman D, Danne TP (2004). ESPE/LWPES consensus statement on diabetic ketoacidosisin children and adolescents. Arch Dis Child.

[10] Smith CP, Firth D, Bennett S, Howard C, Chisholm P (1998). Ketoacidosis occurring in newly diagnosed and established diabetic children. Acta Paediatr.

[11] Rewers A, Klingensmith G, Davis C, Petitti DB, Pihoker C, Rodriguez B (2008). Presence of diabeteic ketoacidosis at diagnosis of diabetes mellitus in youth: the search for Diabetes in Youth Study. Pediatrics.

[12] Umpierrez GE, Kitabchi AE (2003). Diabetic ketoacidosis: risk factors and management strategies. Treat Endocrinol.

[13] Bui H, To T, Stein R, Fung K, Daneman D (2010). Is Diabetic Ketoacidosis at Disease Onset a Result of Missed Diagnosis?. J Pediatr.

[14] Dumont RH, Jacobson AM, Cole C, Hauser ST, Wolfsdorf JI, Willett JB (1995). Psychosocial predictors of acute complications of diabetes in youth. Diabet Med.

[15] Bryden KS, Neil A, Mayou RA, Peveler RC, Fairburn CG, Dunger DB (1999). Eating habits, body weight and insulin misuse. A longitudinal study of teenagers and young adults with type 1 diabetes. Diabetes Care.

[16] Mortensen HB, Swift PG, Holl RW, Hougaard P, Hansen L, Bjoerndalen H (2010). Multinational study in children and adolescents with newly diagnosed type 1 diabetes: association of age, ketoacidosis, HLA status, and autoantibodies on residual beta-cell function and glycemic control 12 months after diagnosis. Pediatr Diabetes.

[17] Vanelli M, Scarabello C, Fainardi V (2008). Available tools for primary ketoacidosis prevention at diabetes diagnosis in children and adolescents. “The Parma campaign”. Acta Biomed.

